# Hepcidin-25 in Chronic Hemodialysis Patients Is Related to Residual Kidney Function and Not to Treatment with Erythropoiesis Stimulating Agents

**DOI:** 10.1371/journal.pone.0039783

**Published:** 2012-07-13

**Authors:** Neelke C. van der Weerd, Muriel P. C. Grooteman, Michiel L. Bots, Marinus A. van den Dorpel, Claire H. den Hoedt, Albert H. A. Mazairac, Menso J. Nubé, E. Lars Penne, Carlo A. Gaillard, Jack F. M. Wetzels, Erwin T. Wiegerinck, Dorine W. Swinkels, Peter J. Blankestijn, Piet M. ter Wee, CONTRAST investigators

**Affiliations:** 1 Department of Nephrology, VU Medical Center, Amsterdam, The Netherlands; 2 Department of Nephrology, Academic Medical Center, University of Amsterdam, Amsterdam, The Netherlands; 3 Institute for Cardiovascular Research VU Medical Center (ICaR-VU), VU Medical Center, Amsterdam, The Netherlands; 4 Julius Center for Health Sciences and Primary Care, University Medical Center Utrecht, Utrecht, The Netherlands; 5 Department of Internal Medicine, Maasstad Hospital, Rotterdam, The Netherlands; 6 Department of Nephrology, University Medical Center Utrecht, Utrecht, The Netherlands; 7 Department of Nephrology, Radboud University Nijmegen Medical Center, Nijmegen, The Netherlands; 8 Department of Laboratory Medicine, Laboratory of Genetic, Endocrine and Metabolic Diseases, Radboud University Nijmegen Medical Center, Nijmegen, The Netherlands; 9 Hepcidinanalysis.com, Radboud University Nijmegen Medical Center, Nijmegen, The Netherlands; University of Florida, United States of America

## Abstract

Hepcidin-25, the bioactive form of hepcidin, is a key regulator of iron homeostasis as it induces internalization and degradation of ferroportin, a cellular iron exporter on enterocytes, macrophages and hepatocytes. Hepcidin levels are increased in chronic hemodialysis (HD) patients, but as of yet, limited information on factors associated with hepcidin-25 in these patients is available. In the current cross-sectional study, potential patient-, laboratory- and treatment-related determinants of serum hepcidin-20 and -25, were assessed in a large cohort of stable, prevalent HD patients. Baseline data from 405 patients (62% male; age 63.7±13.9 [mean SD]) enrolled in the CONvective TRAnsport STudy (CONTRAST; NCT00205556) were studied. Predialysis hepcidin concentrations were measured centrally with matrix-assisted laser desorption/ionization time-of-flight mass spectrometry. Patient-, laboratory- and treatment related characteristics were entered in a backward multivariable linear regression model. Hepcidin-25 levels were independently and positively associated with ferritin (p<0.001), hsCRP (p<0.001) and the presence of diabetes (p = 0.02) and inversely with the estimated glomerular filtration rate (p = 0.01), absolute reticulocyte count (p = 0.02) and soluble transferrin receptor (p<0.001). Men had lower hepcidin-25 levels as compared to women (p = 0.03). Hepcidin-25 was not associated with the maintenance dose of erythropoiesis stimulating agents (ESA) or iron therapy. In conclusion, in the currently studied cohort of chronic HD patients, hepcidin-25 was a marker for iron stores and erythropoiesis and was associated with inflammation. Furthermore, hepcidin-25 levels were influenced by residual kidney function. Hepcidin-25 did not reflect ESA or iron dose in chronic stable HD patients on maintenance therapy. These results suggest that hepcidin is involved in the pathophysiological pathway of renal anemia and iron availability in these patients, but challenges its function as a clinical parameter for ESA resistance.

## Introduction

Hepcidin is a key regulator of iron homeostasis in humans. It induces internalization and degradation of ferroportin, which is a cellular iron exporter on enterocytes, macrophages and hepatocytes [1], [2]. Hence, hepcidin reduces iron absorption from the gut and iron release from reticuloendothelial and hepatocyte stores. The bioactive form is hepcidin-25, a mainly protein-bound amino acid of 2.8 kD, whereas hepcidin-20 and hepcidin-22 are its isoforms with unknown biological function [2], [3]. The expression of hepcidin is regulated in response to iron administration, erythropoietic demand, hypoxia and inflammatory signals [2], [4].

Hepcidin is excreted with the urine. In patients with chronic kidney disease (CKD), serum levels of the active hepcidin-25 and its isoforms are increased [5], [6]. In patients with end stage renal disease (ESRD) on dialysis, even higher levels of hepcidin have been observed [5], [6]. Hepcidin is the intermediary between available iron stores on the one hand, and erythropoiesis on the other hand. Furthermore, it has been suggested that hepcidin is an important tool to predict the response to erythropoiesis stimulating agents (ESA) [7], [8], [9]. Therefore, hepcidin might be useful to assess the functional iron availability in patients with renal failure as high levels might indicate a blockade of iron release from its stores [10].

In several studies, patient-, laboratory- and treatment characteristics of CKD and ESRD patients have been related with hepcidin levels. Many studies have shown a relation between ferritin levels and hepcidin, both in CKD [5], [6], [9], [11] and in hemodialysis (HD) patients [5], [12], [13], [14], [15]. Furthermore, studies in CKD and HD patients have shown associations with hepcidin and various other parameters such as residual kidney function (RKF) [6], [11], [16], ESA dose [11] and markers of inflammation including C-reactive protein (CRP), tumor necrosis factor α (TNF-α) and interleukin-6 (IL-6) [7], [15]. In these studies, hepcidin has been measured with different techniques, mainly competitive immunoassays and mass spectrometry (MS) based methods, impeding direct comparisons [3], [17], [18]. Furthermore, most studies on hepcidin in HD patients included a limited number of patients, precluding multivariate statistics.

In the current study, patient-, laboratory- and treatment characteristics that are associated with hepcidin levels are evaluated with a state-of-the-art hepcidin assay in a prospective cohort of over 400 chronic HD patients, included in the CONvective TRAnsport STudy (CONTRAST).

## Materials and Methods

### Patients and Study Design

Baseline data from patients enrolled in the CONTRAST study (NCT00205556) were used. The rationale and the design of the CONTRAST study have been described before [19]. In short, prevalent HD patients were recruited from 2004 until 2010 and randomized to either continue treatment with low flux HD, or switch to treatment with post-dilution online hemodiafiltration, both with ultrapure dialysate, with a variable follow up until December 2010. Primary endpoint of the study is all cause mortality [20], and anemia management is a secondary endpoint. A total of 714 patients were recruited from 29 dialysis centers. In the design phase of CONTRAST, a protocol for blood sampling and storage was added, specifically for future studies on newly identified markers that would become potentially relevant and of interest. Hepcidin is an example of such a marker. In 17 of the 29 dialysis centers, in which blood sampling and storage was logistically feasible, predialysis blood samples from participating patients were drawn and stored at −80°C. The selection of centers participating in this sub-study was made prior to enrolment. The present analyses were based on a subset of patients from the main study, namely those participants (n = 405) from who additional blood samples were collected.

Patients were eligible for inclusion in the main study if they were treated two or three times per week with HD for at least two months. Exclusion criteria were age below 18 years, treatment with hemo(dia)filtration or high-flux HD in the six months prior to randomization, a life expectancy less than three months due to non-renal disease, participation in another clinical intervention trial evaluating cardiovascular outcomes and severe incompliance regarding frequency and/or duration of dialysis treatment.

The study was conducted in accordance with the Declaration of Helsinki and was approved by a central medical ethics committee and by all local medical ethics review boards. Written informed consent was obtained from all patients prior to enrolment (File S1). Patients provided informed consent for storage of blood samples for later analysis.

### Treatment Protocol

Included patients were stable for at least two months with a minimum dialysis spKt/V_urea_ of 1.2 per treatment and they were treated with either polysulfone (PS) or polyarylethersulfone (PAES) low-flux dialyzers with a UF coefficient varying between 10 and 21 ml/mmHg/h and a surface area from 1.3 to 2.2 m^2^: F6HPS, F7HPS, F8HPS and F10HPS (Fresenius Medical Care, Bad Homburg, Germany) and Polyflux 14 L, 17 L and 21 L (Gambro Corporation AB, Lund, Sweden). Dialysis was performed with ultrapure dialysis fluids, containing less than 0.1 colony forming units per mL and less than 0.03 endotoxin units per mL.

Routine patient care and prescription of medication was practiced according to the opinion of the attending nephrologist and based on the Quality of Care Guidelines of the Dutch Federation of Nephrology. The Dutch Quality of Care Guideline on anemia management was derived from the European Best Practice Guidelines [21] and the KDOQI guidelines [22], [23], [24]. ESA and iron supplements were administered via the venous bloodline at the end of a dialysis session. Decisions on dose changes and the timing of these changes were made according to the opinion of the treating nephrologist.

### Laboratory Protocol

Predialysis blood samples were drawn and routine laboratory assessments were analyzed in the local hospitals by standard laboratory techniques. The total iron-binding capacity (TIBC) was considered to represent serum transferrin level [25] and the transferrin saturation ratio (TSAT) was either provided by the local laboratory or calculated as serum iron divided by the TIBC. Hepcidin, soluble transferrin receptor (sTfR), hsCRP and IL-6 measurements were preformed centrally. For this purpose, predialysis blood samples were centrifuged at 1500 g and 4°C for 10 minutes and stored at −80°C.

Serum hepcidin-20 and -25 measurements were centrally performed by a validated combination of weak cation exchange (WCX) bead-based hepcidin enrichment followed by time-of-flight mass spectrometry (WCX-TOF-MS) [17]. For the quantification of hepcidin in serum, an internal standard (synthetic hepcidin-24, Peptide International Inc., Louisville, KY, USA) was used [26]. Peptide spectra were generated on a Microflex LT matrix-enhanced laser desorption/ionisation (MALDI-) TOF-MS platform (Bruker Daltonics GmbH, Bremen, Germany). Serum hepcidin-20 and -25 concentrations were expressed as nmol/L and the lower limit of detection of this method was 0.5 nmol/L. For hepcidin-25, the intra-assay coefficients of variation (CV) were 3.7% at 7.9 nmol/L, 2.3% at 13.4 nmol/L, and 2.2% at 3.1 nmol/L. The inter-assay CV were 9.1% at 7.8 nmol/L and 3.9% at 12.9 nmol/L [17]. This method enables the specific measurement of the hepcidin isoforms (hepcidin-25, hepcidin-22 and hepcidin-20) [17] and has been described before in CKD and HD patients [5]. It is an update of a previous method performed by the same laboratory [26], [27]. sTfR (mg/L) was measured immunonephelometrically on a BN II System (Dade Behring Marburg GmbH, Marburg, Germany). hsCRP (mg/L) was measured with a particle-enhanced immunoturbidimetric assay on a Roche-Hitachi analyzer (Roche Diagnostics GmbH, Mannheim, Germany) with a lower quantification limit of 0.1 mg/L and an intra-assay variation of 1.9% at the level of 0.57 mg/L and 0.3% at the level of 3.00 mg/L. The inter-assay variation was 1.9% at the level of 0.67 mg/L and 1.2% at the level of 3.64 mg/L. IL-6 (pg/mL) was measured with an immunometric assay (Sanquin, Amsterdam, The Netherlands). The intra-assay variation was 12% at the level of 1 pg/mL and 8% at the level of 3 pg/mL. The inter-assay variation was 19% at the level of 0.35 pg/mL (which was the lower quantification limit) and 12% at the level of 2.3 pg/mL.

### Data Collection

Data on demography, cause of renal failure, history of cardiovascular disease (CVD), diabetes mellitus (DM), type of vascular access, dialysis vintage and treatment parameters were collected, as well as medication use. ESA was prescribed as epoetin α or β (Eprex® or Neorecormon® respectively, IU) or darbepoetin α (Aranesp®, µg) and expressed as a dose per week. To compare the different types of ESA, prescribed dosages were converted to daily defined doses (DDD), using conversion factors as provided by the World Health Organization (WHO) Drug Classification (http://www.whocc.no/atc_ddd_index/). For darbepoetin α (ATC code B03XA02), DDD is 4.5 µg and for epoetin α and β (ATC code B03XA01), DDD is 1000 IU. All patients on iron therapy used iron sucrose (Venofer®, mg/week).

RKF was defined as a urine production of >100 mL/d. In patients with RKF, the eGFR (estimated glomerular filtration rate) was calculated as the mean of creatinine and urea clearance in a 24 h urine collection, adjusted for body surface area [28].

### Statistical Analysis

Variables were reported as proportions or means ± standard deviation (SD), or medians with 25^th^–75^th^ percentiles when appropriate. The relation between hepcidin-20 and hepcidin-25 was evaluated with a Spearman’s correlation test. All patient characteristics, laboratory parameters and treatment characteristics listed in table 1, were considered as possible determinants of hepcidin-25. First, relations between these determinants and hepcidin-25 were studied using a backward multivariable linear regression model with a p-value <0.15 as a cut-off level. Subsequently, the determinants that were related with Hepcidin-25 (with a p-value <0.15) were entered in a second multivariable regression model. In this second regression model, a double sided p-value <0.05 was considered statistically significant. The natural logarithm of hepcidin-25 (ln-hepcidin-25) was applied as the dependent variable in all regression models since the distribution of hepcidin-25 was positively skewed. The regression coefficients (B) were retransformed into percentages of change in hepcidin-25 by using the formula (e^B^-1)×100, which means that for each increment in the determinant, hepcidin-25 changed with the specified percentage. Additionally, in a separate analysis, all regression models were adjusted for participating center to correct for local policies concerning anemia management and timing of ESA and iron administration and blood withdrawal.

**Table 1:** Patient and treatment characteristics and laboratory parameters.^a^

	N = 405
***Patient characteristics***	
Male gender – no. (%)	252 (62)
Age (years)	63.7±13.9
Caucasian race – no. (%)	333 (82)
Dialysis vintage (years)	1.8 (0.9–3.6)
Cause of renal failure - no. (%)	
- vascular	131 (32)
- diabetes mellitus	63 (16)
- tubulointerstitial nephritis/glomerulo-nephritis/multisystem disease	96 (24)
- cystic disease	28 (7)
- other/unknown	87 (21)
Diabetes mellitus – no. (%)	85 (21)
History of cardiovascular disease – no. (%)	177 (44)
Current smoker – no. (%)	81 (20)
Body weight (kg)^b^	71.7±14.6
Systolic blood pressure (mmHg)^c^	142±18
Diastolic blood pressure (mmHg)^c^	73±11
BMI (kg/m^2^)	25.0±4.8
Residual diuresis – no. (%)^d^	230 (57)
eGFR (ml/min/1.73 m^2^)^e^	2.6 (1.2–5.1)
***Treatment characteristics***	
Treatment frequency 3x/week – no. (%)	375 (93)
Treatment time (min)	227±23
Bloodflow (mL/min)	298±39
Dialysis access – no. (%)	
- fistula	339 (84)
- graft	56 (14)
- central catheter	10 (2)
spKt/V (per dialysis)	1.39±0.20
Dialyzer – no. (%)	
- polysulfone	246 (61)
- polyarylethersulfone	147 (37)
- other	12 (3)
Prescription of ESA- no. (%)	364 (90)
Type of ESA – no. (%)	
- darbepoetin α	254 (70
- epoetin α/β	110 (30)
ESA dose (DDD/week)^f^	8.9 (6.0–15.4)
Use of iron replacement therapy – no. (%)	300 (74)
Irondose (mg/week)^g^	100 (50–100)
Prescription of RAS inhibitors – no. (%)	215 (53)
Prescription of statin – no. (%)	203 (50)
***Laboratory parameters***	
Hemoglobin (g/dL)	11.9±1.3
Hematocrit	0.36±0.04
MCV (fl)	94.9±6.2
Reticulocytes (x10^9^/L)	65.3±30.5
Ferritin (ng/mL)	378 (211–631)
TSAT (%)	24.3±12.4
sTfR (mg/L)^h^	1.58 (1.24–2.11)
Cholesterol (mg/dL)	143.1±38.7
Albumin (g/dL)	3.6±0.5
hsCRP (mg/L)	3.95 (1.38–10.41)
Il-6 (pg/mL)	2.06 (1.21–3.82)
Hepcidin-20 (nM)	6.3 (3.9–9.3)
Hepcidin-25 (nM)^i^	13.8 (6.6–22.5)

a Values represent mean ± SD, median (interquartile range) or proportion (%).

b Weight after dialysis (dry weight) defined as the mean of three consecutive values.

c Mean of pre- and post-dialysis blood pressure of three consecutive dialysis sessions.

d Defined as >100 mL per day.

e eGFR (estimated glomerular filtration rate) calculated as mean of creatinine and urea clearance in 24 h urine collection adjusted for body surface area, exclusively in patients with residual diuresis.

f In patients on ESA therapy.

g In patients on iron therapy.

h Reference value: 0.76–1.76 mg/L (Dade Behring Marburg GmbH, Marburg, Germany).

i Reference value (median [95% range]): men 65–69 years 5.3 (<0.05–13.9); women 65–69 years 4.9 (<0.05–14.2) [29].

Conversion factors for units: hemoglobin in g/dL to mmol/L, x 0.62; cholesterol in mg/dL to mmol/L, x 0.026; albumin in g/dL to g/L, x 10; no conversion necessary for ferritin in ng/mL to µg/L.

BMI = body mass index; ESA = erythropoiesis stimulating agents; RAS = renin angiotensin system; TSAT = transferrin saturation ratio; sTfR = soluble transferrin receptor; PTH = parathyroid hormone; hsCRP = high sensitive c-reactive protein; IL-6 = interleukin-6.

To evaluate whether the relation between a determinant (e.g. hsCRP) and hepcidin-25 was modified by a second determinant (e.g. ferritin), the possibility of effect modification was explored by adding an interaction term (e.g. hsCRP x ferritin) to the multivariable regression model. If this interaction term turned out to be significant (p<0.05), the relation between the determinant and hepcidin-25 was analyzed separately in each stratum of the second determinant.

Statistical analyses were performed with PASW software (version 18.0, SPSS inc. Headquarters, Chicago, Illinois, US).

## Results

**Figure 1:**
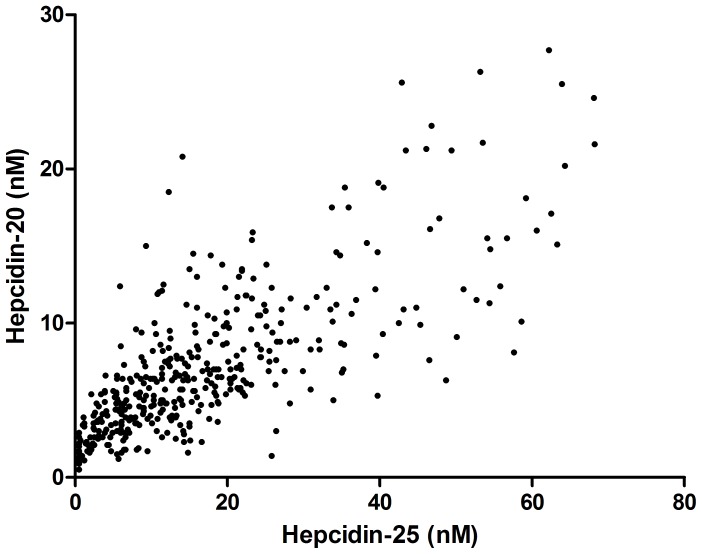
Correlation of Hepcidin-25 with its isoform hepcidin-20. Hepcidin-20 and -25 were measured with mass spectrometry (WCX- MALDI-TOF-MS, see section on laboratory protocol). r = 0.76; p-value <0.001.

Blood samples from 405 patients were available. All patient and treatment characteristics and laboratory parameters are listed in table 1 (baseline characteristics of the total CONTRAST cohort [n = 714] are listed in table S1). Mean (± SD) age of the patients was 63.7±13.9 years and 62% was male. Hepcidin-20 and hepcidin-25 were highly correlated (r = 0.76; p<0.001; figure 1). In this section, results for hepcidin-25 are presented. In analyses with hepcidin-20 as an outcome parameter, similar results were obtained (data not shown).

### Multivariable Regression Analysis

In table 2, all determinants of hepcidin showing a p-value <0.15 in the backward multivariable linear regression model are listed. In the final model (R2 = 0.49), ferritin, hsCRP and the presence of diabetes mellitus showed a positive relation with hepcidin-25, whereas male gender, eGFR and sTfR had an inverse relation. Of note, no relation between hepcidin-25 and the weekly ESA dose and the administration of iron supplements was observed. Adjustment for participating center did not change the results (data not shown).

**Table 2:** Results from the multivariable regression analysis on hepcidin-25 levels.^a^

	Multivariable regression				
Determinant	B^b^	95% CI^b^	% change^c^	95% CI^c^	P-value
Gender (male)	−0.188	−0.361 to −0.016	−17.1	−30.3 to −1.6	0.032
Diabetes	0.246	0.034 to 0.458	27.9	3.5 to 58.1	0.023
Current smoker	−0.188	−0.390 to 0.014	−17.1	−32.3 to 1.4	0.067
Prescription of statins	−0.162	−0.332 to 0.009	−15.0	−28.3 to 0.9	0.063
Prescription of RAS inhibitors	0.113	−0.056 to 0.282	12.0	−5.4 to 28.7	0.053
eGFR (per mL/min/1.73 m^2^)	−0.033	−0.057 to −0.008	−3.2	−5.5 to −0.5	0.008
Hemoglobin (per g/dL)	0.085	0.019 to 0.152	8.9	1.9 to 16.4	0.012
MCV	−0.011	−0.025 to 0.004	−1.1	−2.5 to 0.4	0.150
Reticulocytes (per 10 *10^9^/L)	−0.034	−0.063 to −0.006	−3.3	−6.1 to −0.6	0.019
Ferritin (per 10 ng/mL)	0.016	0.013 to 0.018	1.6	1.3 to 1.8	<0.001
sTfR (per mg/L)	−0.409	−0.544 to −0.274	−33.6	−42.0 to −24.0	<0.001
hsCRP (per mg/L)	0.012	0.007 to 0.017	1.2	0.7 to 1.4	<0.001

a Regression analyses were performed with natural logarithm of hepcidin-25 as dependent variable. Potential determinants of hepcidin-25 were selected using a backward multivariable linear regression model with a p-value <0.15 used as a cut-off level in which all patient, treatment and laboratory characteristics as listed in table 1 were entered.

b The regression coefficient (B) denotes a natural logarithm. Positive values indicate an increase in hepcidin-25 and negative values a decrease with one unit increase of the determinant.

c Results of conversion of the regression coefficient (B) from natural logarithm to a percentage of change: for each increase in the determinant with one unit, hepcidin-25 changed with the percentage indicated in this column. Positive values indicate an increase in hepcidin-25 and negative values a decrease.

R^2^ for multivariable regression model = 0.49. Further adjustment for participating center did not change the results (data not shown).

### Interaction between Determinants

**Figure 2:**
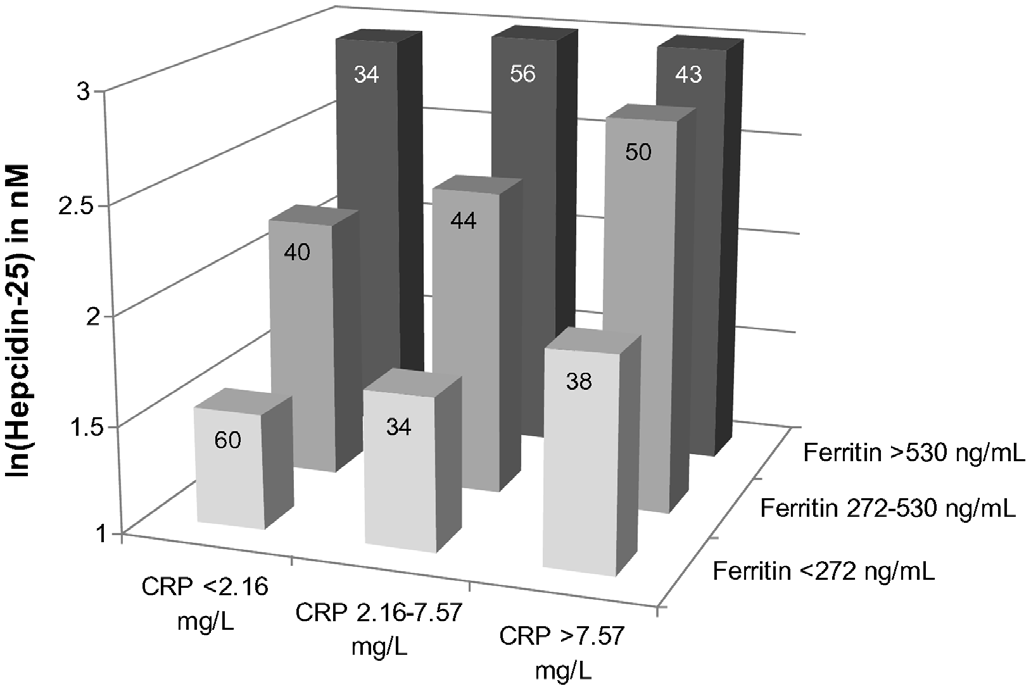
Relationship between ferritin, hsCRP and hepcidin-25. Hepcidin-25 was ln-transformed because of a positively skewed distribution. Values were adjusted for gender, diabetes, smoking status, prescription of statins and RAS inhibitors, eGFR, hemoglobin, MCV, absolute reticulocyte count and the level of soluble transferrin receptor. CRP and ferritin levels were divided in tertiles. Numbers in boxes represent number of patients per category. For 6 patients, ferritin and/or hsCRP levels were missing. P-value for interaction factor (hsCRP x ferritin) <0.001.

The relation between hsCRP and hepcidin-25 was modified by the ferritin level, as the interaction term (hsCRP x ferritin) was highly significant (p<0.001). This relation persisted after adjustment for other determinants of hepcidin-25. As depicted in figure 2, the relation between ferritin and hepcidin-25 was present irrespective of the level of inflammation (lowest hsCRP tertile: B = 0.020 per 10 ng/mL; 95%CI -0.015 to 0.026; p<0.001; middle hsCRP tertile: B = 0.014 per 10 ng/mL; 95%CI 0.010 to 0.018; p<0.001; highest hsCRP tertile: B = 0.015 per 10 ng/mL; 95%CI 0.010 to 0.020; p<0.001). In fact, the relation between hsCRP and hepcidin-25 was present in all three tertiles of ferritin (lowest tertile: B = 0.020 per mg/L; 95%CI −0.010 to 0.030; p<0.001; middle tertile: B = 0.009 per mg/L; 95%CI 0.000 to 0.018; p = 0.055; highest tertile: B = 0.007 per mg/L; 95%CI 0.001 to 0.013; p = 0.034).

**Figure 3:**
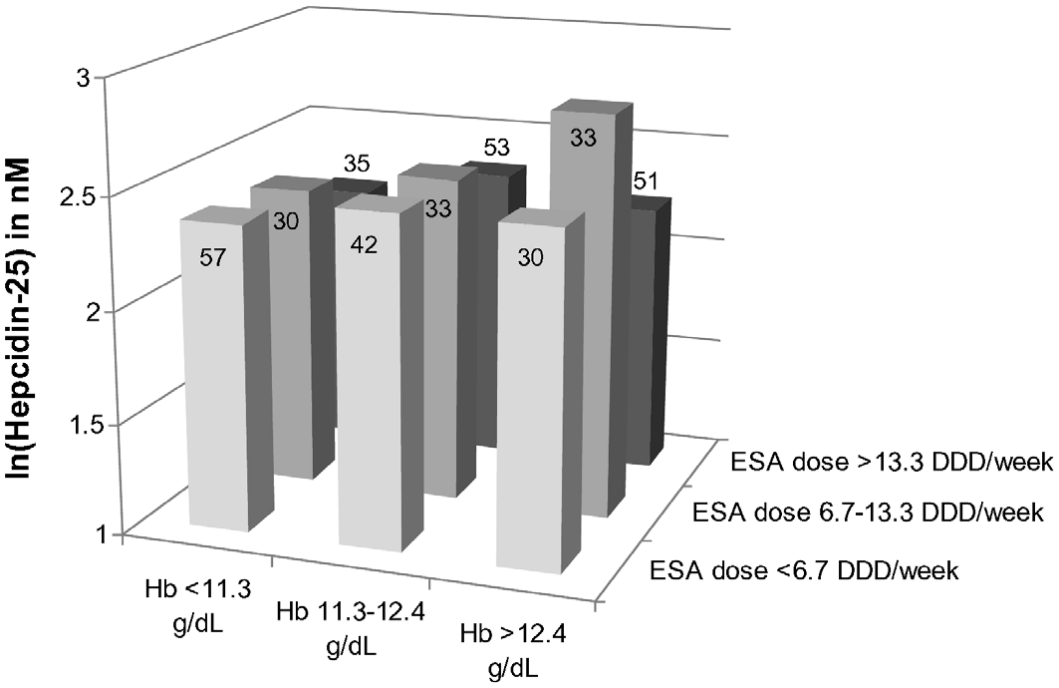
Relationship between ESA dose, hemoglobin and hepcidin-25. Hepcidin-25 was ln-transformed because of a positively skewed distribution. Values were adjusted for gender, diabetes, smoking status, prescription of statins RAS inhibitors, eGFR, MCV, absolute reticulocyte count, ferritin, hsCRP and soluble transferrin receptor. Only patients on ESA therapy are depicted (n = 364). Hemoglobin and ESA dose were divided in tertiles. Numbers in boxes represent number of patients per category. P-value for interaction factor (ESA dose x hemoglobin) NS.

No interaction between hemoglobin level and ESA dose on hepcidin-25 levels was observed as the interaction term (ESA dose X hemoglobin) was not statistically significant (p = 0.588). The absence of a relation between those parameters is readily apparent from figure 3.

## Discussion

In this cross-sectional study in a cohort of stable prevalent HD patients, hepcidin-25 levels were shown to be independently and positively associated with iron stores (as reflected by ferritin levels), inflammation (hsCRP) and the presence of diabetes, and inversely with erythropoiesis (sTfR and reticulocyte count), residual kidney function (eGFR) and male gender. Of note, no relations between hepcidin-25 and either ESA dose or iron supplementation were observed.

In our study, ferritin was the strongest determinant of hepcidin, which has been well established before in healthy controls [29], CKD patients [5], [6], [9], [11] and in patients with ESRD treated with HD and peritoneal dialysis [5], [12], [13], [14], [15]. Notably, the studies in HD patients included mostly low patient numbers. As can be seen from figure 2, the relation between hepcidin-25 and ferritin was present irrespective of the level of inflammation. However, whether hepcidin is upregulated in response to increased ferritin levels cannot be concluded from our study.

sTfR has proven to be a valuable tool to assess bone marrow erythropoietic activity and iron stores in HD patients treated with ESA [30], [31]. However, it could not predict a response of intravenous iron administration on the hemoglobin level [32]. In our study, an inverse association between either sTfR and reticulocyte count, and hepcidin levels was observed, after multivariable adjustments. Whether low hepcidin levels enhance erythropoiesis, or whether increased bone marrow erythropoietic activity suppresses expression of hepcidin, cannot be concluded from this cross-sectional study.

We showed a strong association between hepcidin-25 and hsCRP, but not with IL-6. Several studies have demonstrated a relation between CRP [5], [7], [15], [33] or IL-6 [7], [13] in small groups of chronic HD patients, whereas others did not [14]. The explanation for the association between hepcidin-25 and hsCRP, and not IL-6, is not readily apparent, especially as transcription of hepatic hepcidin is activated by binding of IL-6 to its receptor complex [1]. However, in a murine and human model investigating various factors associated with hepcidin expression, the role of IL-6 was limited [34]. Furthermore, the IL-6 assay used in our study showed a wide inter-assay variability, especially in the lower range. This could have resulted in less accurately measured values of IL-6 as compared to hsCRP, and hence less precision in the estimation.

We are the first to report an independent association between eGFR and both the active hepcidin-25 and the inactive isoform hepcidin-20 in HD patients. As we used a mass-spectrometry assay that specifically measures hepcidin-25, our results indicate that the observed association between eGFR and hepcidin-25 was not due to the concurrent measurement of inactive isoforms. To date, studies on the association between eGFR and hepcidin levels in CKD patients have been conflicting [5], [6], [11], [35]. Low hepcidin levels (measured with a radioimmunoassay) were reported in HD and PD patients with residual diuresis [16], but RKF was not quantified in that study. Whether the high hepcidin levels in chronic HD patients were exclusively caused by decreased renal clearance, or whether other mechanisms are involved, cannot be concluded from our data.

In our study population, hepcidin-25 levels were significantly lower in men as compared to women. This can be explained by the fact that most women in our study will be post-menopausal, in whom higher hepcidin levels have been demonstrated [29]. Furthermore, we showed that diabetic patients had higher hepcidin levels. In one study, diabetic patients had higher levels of hepcidin than healthy age-matched controls, although this relation was not adjusted for possible confounders [36].

Interestingly, we did not observe an interaction between ESA dose and hemoglobin levels in relation to hepcidin-25 as has been demonstrated before by Ashby et al [11]. Therefore, it appears that hepcidin, measured with a mass spectrometric assay in chronic HD patients on maintenance therapy with ESA, is not a marker of ESA resistance. Whether hepcidin-25 can predict an ESA response, as has been shown in patients with the cardio-renal syndrome [9], cannot be concluded from our cross-sectional data. Nevertheless, in a study in 24 HD patients, hepcidin levels of ESA responsive patients did not differ from those who were ESA resistant [12], which is in accordance with our results.

Concerning iron supplementation in HD patients, various effects of iron loading on hepcidin levels have been reported [14], [37], [38]. We did not observe a relationship between hepcidin and iron supplementation, which can be explained by the fact that patients in our study received maintenance iron therapy instead of a (single) loading dose. Recently, it was shown that hepcidin-25 levels did not predict a response to the administration of intravenous iron supplementation in HD patients on ESA maintenance therapy [38]. Hence, it appears that hepcidin is more a marker of iron stores than a predictor of the effect of iron therapy.

A number of studies showed that hepcidin levels could be lowered over a single HD session [13], [15], [39], although concentrations were back to baseline only one hour after the treatment [13]. Lowering of hepcidin by HD can be partly explained by appearance of (low) levels of hepcidin in the ultrafiltrate [5], [16]. In addition, it has been shown that hepcidin can attach to the membrane of the dialyzer [5], which can be explained by the amphipathic and protein-bound structure of hepcidin [2]. Prospective research is needed to draw any conclusions on the effect of different dialyzers on hepcidin-levels.

### Limitations and Strengths

Our study is limited by its cross-sectional design, which impedes assessing causal relationships. Furthermore, a specific treatment protocol for ESA and iron administration and timing of blood sampling was not provided. We tried to compensate for this by adjusting the regression models for participating center in an additional analysis, as the intervals between ESA and/or iron administration and blood sample withdrawal are supposed to be similar within a single treatment center. Since this did not change our results, we conclude that the dosing schedule was not a major confounder in our study. Another potential limitation of our study is the patient selection, based on centers where blood sampling and storage was logistically feasible. This might have introduced a selection bias of which the magnitude and direction cannot be estimated. As selective participation or non-participation must have occurred based on a logistical aspect, which is most probable not related to factors associated with hepcidin or determinants of hepcidin, selection bias seems unlikely.

The strength of our study is the large sample size and the prospective data collection. As far as we know, our study comprises the largest cohort of HD patients in which hepcidin measurements were performed, currently published. The large sample size facilitates multivariable statistics, which is an important method when examining the complex regulation of hepcidin [40]. Moreover, hepcidin measurements have been performed with a validated mass spectrometric technique, enabling specific quantification of the bioactive hepcidin-25.

### Conclusions

In this cohort of chronic, stable HD patients, hepcidin-25 levels were independently associated with iron stores (as reflected by ferritin levels), erythropoiesis (reticulocyte count and sTfR), inflammation (hsCRP), eGFR, the presence of diabetes and gender. Hepcidin-25 was strongly correlated with its bio-inactive isoform hepcidin-20, and similar associations with hepcidin-20 were identified. Of note, hepcidin-25 was not associated with the maintenance dose of ESA or iron therapy.

Our findings confirm the role of hepcidin as a biomarker of iron stores and erythropoiesis in chronic HD patients and indicate that hepcidin is not a biomarker of ESA resistance in patients on ESA maintenance therapy. Furthermore, it underscores the potential important role of (limited) RKF in HD patients. However, whether low hepcidin levels in HD patients are associated with a favorable outcome in terms of morbidity and mortality is not clear yet. Furthermore, whether hepcidin measurements in HD patients provide additional information concerning anemia management compared to current available markers such as ferritin is questionable.

## Supporting Information

File S1 **Patient information and informed consent for the CONTRAST study (original in Dutch, translated in English).**
(DOCX)

Table S1 **Baseline characteristics for entire cohort enrolled in CONTRAST (N = 714) and the Hepcidin cohort (N = 405).**
(DOCX)
