# Supplementary material for: Hepcidin-25 in Chronic Hemodialysis Patients Is Related to Residual Kidney Function and Not to Treatment with Erythropoiesis Stimulating Agents
Source: PLoS One. 2012 Jul 13;7(7):e39783. doi: 10.1371/journal.pone.0039783 (PMC3396629; doi:10.1371/journal.pone.0039783)
Supplement: File S1 — Patient information and informed consent for the CONTRAST study (original in Dutch, translated in English). (DOCX) [file pone.0039783.s001.docx]

**SUPPLEMENTARY FILE 1.**

**Patient information and informed consent for the CONTRAST study (original in Dutch, translated in English).**

**Effect of increased convective clearance by on-line hemodiafiltration on all cause and cardiovascular mortality in chronic hemodialysis patients.**

**The Dutch *CON*vective *TRA*nsport *ST*udy (*CONTRAST*)**

Amsterdam, August 15^th^ 2006

Dear mr/mrs,

Presently, you are being treated with hemodialysis because your kidneys do not function properly any more. The artificial kidney and the dialysis machine purify your blood and remove excess fluid from the body.

There is a continuous effort to improve the quality of hemodialysis treatment. Much energy is put in to efforts to improve the removal of the harmful toxins and waste products from the blood. This concerns specially the substances that are bigger than urea and creatinine, the so called middle molecules. Possibly, these substances play a role in the development of the frequently occurring problems such itching, joint problems and vascular problems due to atherosclerosis.

***Research project***

There is an ongoing trial in a number of dialysis centers in The Netherlands, one center in Norway and one center in Canada that compares two different types of dialysis techniques. Your hospital is also participating in this trial. The two techniques that are compared are standard hemodialysis and so called on-line hemodiafiltration, a newer dialysis technique. This technique removes the toxins and waste products somewhat more efficient, than the standard therapy. Whether this is of any benefit to the patients, is unknown. The primary aim of the study is to compare the effect of these treatments on cardiovascular disease. This will be done by registration of the prevalence of cardiovascular disease, but also by periodically taking blood samples, and by measuring the thickness of the left ventricle of the heart, the stiffness of the aorta, and the thickness of the wall of the carotid artery. Finally, the quality of life will be investigated by a questionnaire.

Until now, you have been treated with standard hemodialysis. If you decide to participate to the study, you will either continue standard hemodialysis or you will be treated with on-line hemodiafiltration. A computer program will determine the type of treatment you will do. Hemodiafiltration has been extensively investigated in earlier trials, and turned out to be safe.

We would like to request you to participate in this trial. In this letter, we wish to inform you about the purpose of the study, the study procedures and the advantages and disadvantages of your participation. After reading this information, you can decide whether you want to participate.

***The study.***

Two different hemodialysis techniques are compared. The first technique is standard hemodialysis. The second is on-line hemodiafiltration. The frequency and length of treatment sessions are identical. The difference between both treatments is that during on-line hemodiafiltration, more middle sized harmful toxins will be removed from the blood. This is done by extracting a larger volume of fluid from the body. Subsequently, a clean, balanced fluid (so called substitution fluid) will be infused. Some people think that the second treatment is better than the first, but there is no scientific evidence to support that. So at this moment, the second treatment is not the standard treatment.

# **Randomization**

In order to make the comparison between the dialysis techniques properly, chance will determine with which technique you will be treated. If you decide to participate, a computer program will choose between the 2 techniques. If you do not wish to participate, you will continue your treatment with standard hemodialysis.

## Course of the trial

Patients who are going to participate to the study, need to be on standard hemodialysis for some months. Then the randomization procedure will be done and chosen therapy will be initiated. This treatment will be continued for 3 years. After those 3 years, we will analyze the results of the two techniques, and then, we can conclude whether the enhanced removal of toxins indeed makes any difference to the patients.

## What does participation for you mean?

You will continue to be treated in your usual schedule, that is two or three times weekly, length of sessions is not changed, During dialysis, blood pressure and heart rate are measured as usual. At the start of the study (after the randomization), a few extra investigations will be done to evaluate the condition of your heart and blood vessels. These investigations will be repeated after 6 months, and after 1,2 and 3 years.

Some information about the investigations:

- *Blood samples*

These are taken from the dialysis blood lines before the start of a session, so no extra venous punctures are necessary. The blood samples include those which are taken routinely as a control of treatment. Some extra samples are taken for measurements of specific substances in the blood, which reflect aspects of the condition of the cardiovascular system. A part of these blood samples will be stored in freezers for later analysis.

- *Ultrasound of carotid and femoral arteries*

We measure the thickness of the carotid artery wall. Further, we measure the velocity of the blood over the aorta. These 2 types of tests are done with ultrasound equipment. During these investigations, that are painless and without side effects, you will be lying on a bed. These investigations will take approximately 50 minutes.

- *Ultrasound of the heart*

During this investigation, you will also be lying on a bed. With an ultrasound machine, images of the structure and function of your heart will be taken and recorded on video. This investigation will take approximately 30 minutes. This investigation is routinely done yearly in dialysis patients. This means, that for this study it will be done one extra time (i.e. six months after the start of the study).

- *Questionnaire for quality of life*

At the start of the study and yearly afterwards, you will be asked to fill in a questionnaire. This will take approximately 20 minutes. If needed, we can help you to do this. The questionnaire contains questions about your daily functioning, your kidney disease and your social environment.

- *Measurement of your nutritional status*

Finally, your nutritional status is measured yearly by registering your dry weight, measuring the thickness of your skin at four different places, measuring the diameter of your upper arm and a short questionnaire. This investigation will take approximately 15 minutes.

Because these investigations will be done yearly (and in the first year also after 6 months), efforts will be made to plan all the investigations on one day.

## Future investigations

It is possible that in the future we will be do studies on genetic material. We wish to request your separate permission on this particular issue already now. DNA is separated from the blood samples. We will analyze whether certain genetic patterns are correlated with the incidence of cardiovascular disease.

The genetic material will be stored, processed and analyzed completely anonymously. If you do not wish to give your permission for this specific part of the study, please indicate so on the Consent Form.

## Side effects

There are no specific side effects to be expected for both treatments, other than you already know for the standard hemodialysis treatment.

## Time for reflection

Obviously, you will need some time to reflect whether you want to participate to this study. You may want to discuss it with other people. Of course, you will have the opportunity for this.

## Confidentiality of the information

The information that will be collected for the study will be processed confidentially. The information will be filled in on specially prepared forms, which are marked with a number instead of your name and personal information. The information will be processed with a code. In scientific publications and presentations, your name will never be used. All the usual procedures to guarantee the protection of your privacy in the hospital will be applied.

To make the study as reliable as possible, it can be necessary to look into your patient file. This can be done by persons that are related to the study, such as a research physician, a research nurse, members of the trial organization, and possibly by representatives of provincial or national authorities (for instance: the Health Inspection). Also members of the Medical Ethical Committee of the hospital can demand access to the study data. They can use this right to investigate the quality of the trial.

## Reimbursement

This study does not come with any costs to you. Part of the extra investigations are considered usual medical care. The other medical investigations are part of medical research, and will not be claimed at your insurance company. In the situation, that you have to visit the hospital specifically for the extra investigations, you may ask for a travel allowance.

## Insurance

This study does not mean any extra health risk to the participants, because it concerns the comparison of two existing and excepted types of therapy. Therefore, the Central Medical Ethical Committee of the Free University Medical Center in Amsterdam has decided that no specific risk insurance is necessary.

## Voluntary participation

Participation in the study is voluntary. At all times, you can withdraw from further participation, even if you have agreed to participate.

This decision will not have any negative influence for your further medical treatment. If your attending physician finds it in your best interest, she/he can decide to stop your participation to the study. He/she will discuss this with you. If you decide not to participate to the study, your regular treatment will be continued.

When new (medical) information will come available during your participation that can influence your decision to participate, you will be informed as soon as possible, so that you can reconsider this decision.

## Further information

If you still have questions after reading this letter, or if you want some further information before or during the study, you can always contact the principal investigator, dr R. Levesque, telephone number .......

## Signing informed consent

If you decide to participate, we ask you to sign a form. Thereby, you confirm your participation to the study. The possibility to stop your participation for any reason remains. Your attending physician will also sign the form as an indication that he/she informed you properly, that you received this information letter, and that he/she is willing to answer any more questions if necessary.

Dr. M.P.C. Grooteman

Department of Nephrology
VU Medical Center

Amsterdam, THE NETHERLANDS

**Informed consent**

**Title of the study:**

Effect of increased convective clearance by on-line hemodiafiltration on all cause and cardiovascular mortality in chronic hemodialysis patients.

(The Dutch CONvective TRAnsport STudy [CONTRAST])

**Responsible physician:** Dr M.P.C. Grooteman

**Hospital:** VU Medical Center, Amsterdam, The Netherlands

I, (name of patient) ..................................................................................................................... hereby declare that I am informed clearly, verbally and in writing, about the aims and methods of the study. My questions have been answered adequately. The written information has been handed over to me.

I agree to participate to the study voluntarily.

I have the right to withdraw my consent without the obligation to give any reason.

I **do / do not** give permission to take blood samples for investigating genetic material (DNA).

Date ........................................... Signature .........................................................................

I, (name of attending physician).....................................................................................................

Have given information about the study verbally and in writing. I declare that I will be willing to answer any further questions about the study if necessary.

Date............................................ Signature.........................................................................
