# Supplementary material for: Hepcidin-25 in Chronic Hemodialysis Patients Is Related to Residual Kidney Function and Not to Treatment with Erythropoiesis Stimulating Agents
Source: PLoS One. 2012 Jul 13;7(7):e39783. doi: 10.1371/journal.pone.0039783 (PMC3396629; doi:10.1371/journal.pone.0039783)
Supplement: Table S1 — Baseline characteristics for entire cohort enrolled in CONTRAST (N = 714) and the Hepcidin cohort (N = 405). (DOCX) [file pone.0039783.s002.docx]

**SUPPLEMENTARY FILE 2**

**Table 1. Baseline characteristics for entire cohort enrolled in CONTRAST (N=714) and the Hepcidin cohort (N=405).**

|  | **Entire cohort (N=714)** | **Hepcidin cohort (N=405)** |
| --- | --- | --- |
| ***Patient characteristics*** | | |
| Male gender – % (no.) | 62 (445) | 62 (252) |
| Age (years) | 64.1 ± 13.7 | 63.7 ± 13.9 |
| Caucasian race – % (no.) | 84 (600) | 82 (333) |
| Dialysis vintage (years) | 2.0 (1.0-4.0) | 1.8 (0.9-3.4) |
| Cause of renal failure - % (no.) |  |  |
| - vascular | 28 (200) | 32 (131) |
| -diabetes mellitus | 19 (136) | 16 (63) |
| - tubulointerstitial nephritis/ glomerulo-  nephritis / multisystem disease | 25 (181) | 24 (96) |
| - cystic disease | 7 (53) | 7 (28) |
| - other/unknown | 21 (144) | 21(87) |
| Diabetes mellitus – % (no.) | 24 (170) | 21 (85) |
| History of cardiovascular disease – % (no.) | 44 (313) | 44 (177) |
| Current smoker – % (no.) | 19 (136) | 20 (81) |
| Body weight (kg)^b^ | 72.4 ± 14.4 | 71.7 ± 14.6 |
| Systolic blood pressure (mmHg)^c^ | 147 ± 21 | 142 ± 18 |
| Diastolic blood pressure (mmHg)^c^ | 75 ± 12 | 73 ± 11 |
| BMI (kg/m^2^) | 25.4 ± 4.4 | 25.0 ± 4.8 |
| Residual diuresis – % (no.)^d^ | 53 (376) | 57 (230) |
| eGFR (ml/min/1.73 m^2^)^e^ | 3.2 (1.3-5.5) | 2.6 (1.2-5.1) |
| ***Treatment characteristics*** | | |
| Treatment frequency 3x/week – % (no.) | 94 (668) | 93 (375) |
| Treatment time (min) | 226 ± 23 | 227 ± 23 |
| Bloodflow (mL/min) | 302 ± 39 | 298 ± 39 |
| Dialysis access – % (no.) |  |  |
| - fistula | 80 (567) | 84 (339) |
| - graft | 14 (100) | 14 (56) |
| - central catheter | 6 (47) | 2 (10) |
| spKt/V (per dialysis) | 1.41 ± 0.24 | 1.39 ± 0.20 |
| Dialyzer – % (no.) |  |  |
| - polysulfone | 62 (439) | 61 (246) |
| - polyarylethersulfone | 36 (260) | 37 (147) |
| - other | 2 (15) | 3 (12) |
| Prescription of ESA- % (no.) | 89 (633) | 90 (364) |
| Type of ESA – % (no.) |  |  |
| - darbepoetin α | 72 (458) | 70 (254) |
| - epoetin α/β | 28 (175) | 30 (110) |
| ESA dose (DDD/week)^f^ | 8.9 (4.4-13.3) | 8.9 (6.0-15.4) |
| Use of iron replacement therapy – % (no.) | 66 (471) | 74 (300) |
| Irondose (mg/week)^g^ | 50 (23-100) | 100 (50-100) |
| Prescription of RAS inhibitors – % (no.) | 49 (349) | 53 (215) |
| Prescription of statin – % (no.) | 32 (231) | 33 (134) |
| ***Laboratory parameters*** | | |
| Hemoglobin (g/dL) | 11.8 ± 1.3 | 11.9 ± 1.3 |
| Hematocrit | 0.36 ± 0.04 | 0.36 ± 0.04 |
| MCV (fl) | 94.8 ± 6.2 | 94.9 ± 6.2 |
| Reticulocytes (x10^9^/L) | 65.6 ± 31.3 | 65.3 ± 30.5 |
| Ferritin (ng/mL) | 339 (193-587) | 378 (211-631) |
| TSAT (%) | 24.1 ± 11.4 | 24.3 ± 12.4 |
| sTfR (mg/L)^h^ | NA | 1.58 (1.24-2.11) |
| Cholesterol (mg/dL) | 141.4 ± 36.8 | 143.1 ± 38.7 |
| Albumin (g/dL) | 3.7 ± 0.5 | 3.6 ± 0.5 |
| hsCRP (mg/L) | NA | 3.95 (1.38-10.41) |
| Il-6 (pg/mL) | NA | 2.06 (1.21-3.82) |
| Hepcidin-20 (nM) | NA | 6.3 (3.9-9.3) |
| Hepcidin-25 (nM)^i^ | NA | 13.8 (6.6-22.5) |

^a^Values represent mean ± SD, median (interquartile range) or % (absolute numbers).

^b^Weight after dialysis (dry weight) defined as the mean of three consecutive values

^c^Mean of pre-dialysis blood pressure of three consecutive dialysis sessions.

^d^Defined as >100 mL per day

^e^eGFR (estimated glomerular filtration rate) calculated as mean of creatinine and urea clearance in 24h urine collection adjusted for body surface area, exclusively in patients with residual renal function.

^f^In patients on ESA therapy.

^g^In patients on iron therapy.

^h^Reference value: 0.76-1.76 mg/L (Dade Behring Marburg GmbH, Marburg, Germany).

^i^Reference value with WCX-TOF MS method (median [95% range]): men 65-69 years 5.3 (<0.05-13.9); women 65-69 years 4.9 (<0.05-14.2).
